# Hesperidin Produces Cardioprotective Activity via PPAR-γ Pathway in Ischemic Heart Disease Model in Diabetic Rats

**DOI:** 10.1371/journal.pone.0111212

**Published:** 2014-11-04

**Authors:** Yogeeta O. Agrawal, Pankaj Kumar Sharma, Birendra Shrivastava, Shreesh Ojha, Harshita M. Upadhya, Dharamvir Singh Arya, Sameer N. Goyal

**Affiliations:** 1 School of Pharmaceutical Sciences, Jaipur National University, Jagatpura, Jaipur, Rajasthan, India; 2 Department of Pharmacology, All India Institute of Medical Sciences, New Delhi, India; 3 Department of Pharmaceutics and Pharmacology, R. C. Patel Institute of Pharmaceutical Education and Research, Shirpur, Maharashtra, India; 4 Department of Pharmacology and Therapeutics, College of Medicine and Health Sciences, United Arab Emirates University, Al-Ain, United Arab Emirates; 5 Sunil's Daibetes and Research Center, Nagpur, Maharashtra, India; Virginia Commonwealth University, United States of America

## Abstract

The present study investigated the effect of hesperidin, a natural flavonoid, in cardiac ischemia and reperfusion (I/R) injury in diabetic rats. Male Wistar rats with diabetes were divided into five groups and were orally administered saline once daily (IR-sham and IR-control), Hesperidin (100 mg/kg/day; IR-Hesperidin), GW9962 (PPAR-γ receptor antagonist), or combination of both for 14 days. On the 15^th^ day, in the IR-control and IR-treatment groups, rats were subjected to left anterior descending (LAD) coronary artery occlusion for 45 minutes followed by a one-hour reperfusion. Haemodynamic parameters were recorded and rats were sacrificed; hearts were isolated for biochemical, histopathological, ultrastructural and immunohistochemistry. In the IR-control group, significant ventricular dysfunctions were observed along with enhanced expression of pro-apoptotic protein Bax. A decline in cardiac injury markers lactate dehydrogenase activity, CK-MB and increased content of thiobarbituric acid reactive substances, a marker of lipid peroxidation, and TNF-α were observed. Hesperidin pretreatment significantly improved mean arterial pressure, reduced left ventricular end-diastolic pressure, and improved both inotropic and lusitropic function of the heart (+LVdP/dt and –LVdP/dt) as compared to IR-control. Furthermore, hesperidin treatment significantly decreased the level of thiobarbituric acid reactive substances and reversed the activity of lactate dehydrogenase towards normal value. Hesperidin showed anti-apoptotic effects by upregulating Bcl-2 protein and decreasing Bax protein expression. Additionally, histopathological and ultrastructural studies reconfirmed the protective action of hesperidin. On the other hand, GW9662, selective PPAR-γ receptor antagonist, produced opposite effects and attenuated the hesperidin induced improvements. The study for the first time evidence the involvement of PPAR-γ pathway in the cardioprotective activity of hesperidin in I/R model in rats.

## Introduction

Ischemic heart disease is the leading cause of morbidity and mortality and is predicted to be the major and most common threat to human life by 2020 [1], [2]. Treatments available for myocardial infarction (MI) like ischemic injury targets restoration of blood supply to ischemic tissue and prevent the damage inflicted at the time of injury. While at one hand, imbalance between myocardial blood supply and demand resulting in development of ischemia and induction of necrosis in myocardium results in acute MI [3], oxidative stress produced by generation of free radicals or reactive oxygen species also plays a key role in MI development [4], [5]. Therefore, suppressing free radical generation and/or augmentation of endogenous antioxidant enzymes is reported to limit the infarct size and attenuate myocardial dysfunction [6]. Moreover, diabetic like conditions worsen the complications arise due to the ischemic diseases, and also delays the recovery [7].

Hesperidin (30, 5, 9-dihydroxy-40-methoxy-7-Orutinosyl Flavanone) is an abundant and inexpensive byproduct of Citrus cultivation and isolated from the ordinary orange *Citrus aurantium* and other species of the genus Citrus (family: Rutaceae). It is reported to have antiallergic, radio protective, immuno-modulator, anti-hypertensive and anti-oxidant properties. Administered orally, it is hydrolyzed by intestinal micro flora to yield a major active metabolite hesperidin [8]. Previous studies established its bolstering role in oxidative stress, and considered as safe model for protection against free radicals. There is substantial evidence to suggest that hesperidin exerts protective action in cardiac tissue by its antihypertensive and antioxidant properties [9]. A protective effect of hesperidin against oxidative stress in liver and kidney of diabetic rabbits [10] has also been reported. Some reports evidenced that hesperidin targets peroxisome proliferator-activated receptor-gamma (PPAR-γ) to exert biological actions [11]. PPAR-γ being a member of the ligand-dependent nuclear receptor category regulates glucose, lipid and energy homeostasis [12], [13]. In addition, PPAR-γ regulates cellular proliferation and differentiation inducing apoptosis in a wide spectrum of human tumor cell lines [12], [14]. Various PPAR-γ agonists like pioglitazone have been shown to reduce myocardial injury (infarct size) and inflammation caused by regional myocardial ischemia and reperfusion in rats and rabbits [15]. Therefore, in the present study we attempted to explore the role of hesperidin in cardiac ischemia and reperfusion (I/R) injury in diabetic rats.

Flavonoids like hesperidin are reported to possess satisfactory capability to neutralize free radicals. This antioxidant property may be related to their pharmacological actions and they may be used as protective agents in a number of cardiac diseases. Although the effect of hesperidin on experimentally induced MI by ischemia-reperfusion model has been studied, the mechanisms underlying the effect are yet to be explored. Hence the present study was carried out to investigate the permissive role of PPAR-γ receptors in the cardioprotective activity of hesperidin in diabetic rats using hemodynamic, biochemical, histopathological, ultrastructural and immunohistochemistry in I/R model of MI.

## Materials and Methods

### Animals

The study protocol was reviewed and approved by the Institutional Animal Ethics Committee, All India Institute of Medical Sciences, New Delhi, India and conformed to the Indian National Science Academy (INSA) Guidelines for the use and care of experimental animals in research. Male Wistar rats weighing 150–250 g were obtained from the Central Animal House Facility of All India Institute of Medical Sciences, New Delhi, India. The rats were maintained under standard laboratory conditions at 25±2°C, relative humidity 60±15% and natural light-dark photo period. Commercial pellet diet (Ashirwad Industries Ltd., Chandigarh, India) and tap water were provided *ad libitum*. The commercial pellet diet contained 24% protein, 5% fat, 4% fibre, 55% carbohydrate, 0.6% calcium, 0.3% phosphorous, 10% moisture, and 9% ash w/w.

### Chemicals

Hesperidin was a purchased from Calbiochem, USA. Creatine kinase (CK-MB) isoenzyme detection kit was purchased from Logotech India Pvt. Ltd. (Delhi, India). The ABC staining kit and primary (Bax mouse monoclonal IgG2b and Bcl-2 mouse monoclonal IgGI) and secondary antibodies (anti-mouse IgG) were procured from Santa Cruz Biotechnology, USA. All chemicals were of analytical grade, purchased from Sigma Chemical Co., St. Louis, USA. Double distilled water was used in all biochemical assays and molecular techniques.

### Induction of Diabetes

**Figure 1:**
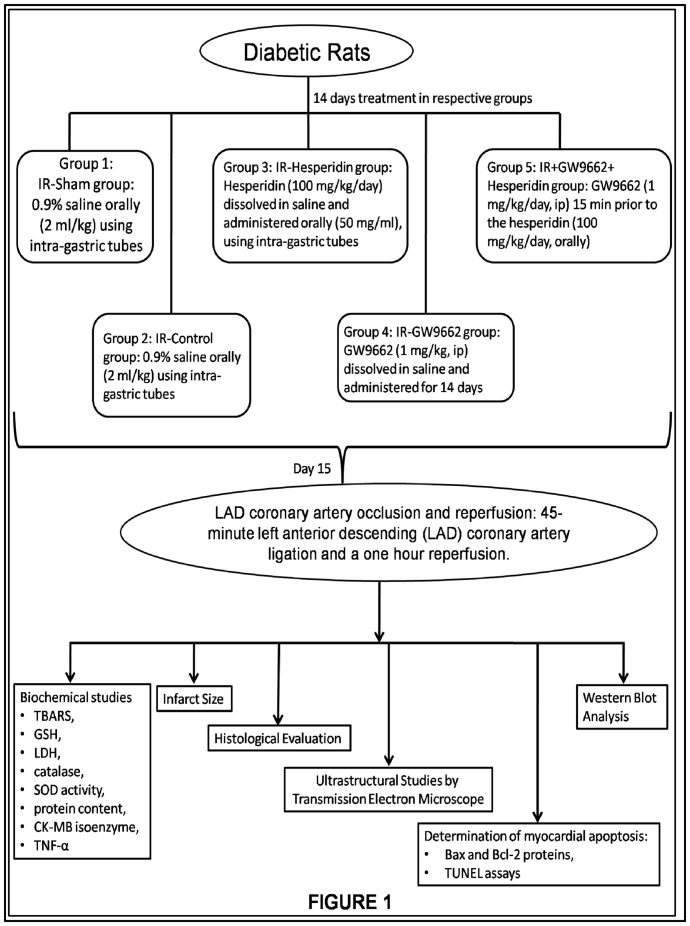
Schematic representation of several groups used in the study showing the treatments received by the diabetic rats for 14 days. On day 15^th^ these rats were subjected to the LAD coronary artery ligation followed by a one hour reperfusion induced myocardial injury. Moreover, these groups were assessed for several biochemical parameters, histological evaluations, ultrastructural studies, myocardial apoptosis markers and western blot analysis.

Streptozotocin (50 mg/kg, 0.1 M cold citrate buffer, pH 4.5) was administered by intraperitoneal (i.p.) route to induce diabetes in rats and was confirmed 3 days post injection by glucoseoxidase/peroxidase method. Briefly, Diabetes was induced by a single intraperitoneal injection of STZ (50 mg/kg body weight) after overnight fasting. Control rats were injected with vehicle buffer only. Blood samples were obtained from the tail vein 72 hr after STZ injection and blood glucose levels were determined. Here in, glucose is oxidized by glucose oxidase (GOD) to produce gluconate and hydrogen peroxide. In the presence of peroxidase (POD), the hydrogen peroxide is then oxidatively coupled with 4 amino- antipyrene (4-AAP) and phenol to yield a red quinoeimine dye that is measured at 505 nm. The absorbance at 505 nm is proportional to concentration of glucose in the sample. Blood glucose level >250 mg/dl was considered as an index of successful induction of diabetes and such rats showing this value were included in the study. Upon confirmation for diabetes, rats were randomly divided into five groups containing 12 rats in each group and used for further experiments (Figure 1).

### Experimental protocol

The animals were randomly divided into five main groups as depicted in Figure 1.

#### Group 1: IR-sham group

Diabetic rats were administered 0.9% saline orally (2 ml/kg) using intra-gastric tubes for 14 days and then sacrificed on the 15^th^ day. The animals were subjected to the entire surgical procedure and thread was passed beneath the coronary artery, but the left anterior descending (LAD) coronary artery was not ligated. The number of animals studied in this group was 12.

#### Group 2: IR-control group

In this group, 12 diabetic rats were administered 0.9% saline orally (2 ml/kg) using intra-gastric tubes for 14 days; thereafter, on the 15^th^ day, the experimental animals were subjected to a 45-minute LAD coronary artery ligation followed by a one hour reperfusion induced myocardial injury.

#### Group 3: IR-hesperidin group

Hesperidin (100 mg/kg/day) was dissolved in saline and was administered orally (50 mg/ml), using intra-gastric tubes, to additional 12 animals for 14 days. On the 15^th^ day, the rats were subjected to a protocol of a 45-minute LAD coronary artery ligation and a one-hour reperfusion.

#### Group 4: IR-GW9662 group

In another group of 12 rats, GW9662 (1 mg/kg, ip) was dissolved in saline and was administered to animals for 14 days. On the 15^th^ day, the rats were subjected to a protocol of a 45-minute LAD coronary artery ligation and a one hour reperfusion.

#### Group 5: IR+GW9662+hesperidin group

In this group, 12 rats were treated with GW9662 (1 mg/kg/day, ip) 15 min prior to the hesperidin (100 mg/kg/day, orally) for 14 days. On the 15^th^day, the rats were subjected to a 45-minute LAD coronary artery ligation followed by one hour reperfusion.

### Experimental procedure for LAD coronary artery occlusion and reperfusion

Following treatments all these groups were subjected to LAD coronary artery occlusion-reperfusion and recording of haemodynamic parameters as described previously [7]. Briefly, rats of all the experimental groups were anaesthetized with pentobarbitone sodium (60 mg/kg, ip) and maintained with 40 mg/kg doses (given at 60 and 120 mins from first dose). Atropine (0.01 mg/kg, ip) was co-administered with the anaesthetic to reduce broncho-tracheal secretions. Body temperature was monitored and maintained at 37°C throughout the experimental protocol. The neck was opened with a ventral midline incision, and a tracheostomy was performed and the rats were ventilated with room air from a positive pressure ventilator (Inco, India) using compressed air at the rate of 70 strokes per minute and a tidal volume of 10 ml/kg. The left jugular vein was cannulated with a polyethylene tube for continuous infusion of 0.9% saline. After that, the right carotid artery was cannulated and the cannula filled with heparinised saline was connected to the cardiac output monitor CARDIOSYS CO-101 (Experimetria, Hungary) via a pressure transducer for measurement of mean arterial pressure (MAP). A left thoracotomy was performed at the fifth intercostal space and the pericardium was opened to expose the heart. The LAD coronary artery was ligated 4–5 mm from its origin using a 5-0 silk suture with an atraumatic needle, and ends of this ligature were passed through a small vinyl tube to form a snare. After completion of the surgical procedure, the heart was returned to its normal position in the thorax. The thoracic cavity was covered with saline-soaked gauze to prevent the heart from drying. The animals were then allowed to stabilize for 15 minutes before LAD coronary artery ligation. Myocardial ischaemia was induced by one stage occlusion of the LAD coronary artery by pressing the polyethylene tubing against the ventricular wall and then fixing it in place by clamping the vinyl tube with a haemostat (except in IR-sham rats). A wide bore (1.5 mm) sterile metal cannula was inserted into the cavity of the left ventricle from the posterior apical region of the heart. The cannula was connected to a pressure transducer (Gold Statham P23ID, USA) and the whole system was filled with heparinised saline (heparin 50 units/ml). Left ventricular systolic and left ventricular end-diastolic pressure (LVEDP) were measured on a multi channel polygraph (Grass 7D, USA) from the left ventricular pressures curve at lower and higher sensitivity of the preamplifier, respectively. The maximum rates of rise and fall of left ventricular pressure (peak +LVdP/dt and peak –LVdP/dt) were measured by the electronic differentiator from the signal output of the channel recording left ventricular pressure. A bolus of heparin (30 IU) was administered immediately before coronary artery occlusion for prophylaxis against thrombus formation around the snare. The animals then underwent 45 minutes of ischaemia, confirmed visually *in situ* by the appearance of regional epicardial cyanosis. The myocardium was reperfused by releasing the snare gently for a one-hour period. Successful reperfusion was confirmed by visualisation of arterial blood flow through the artery and appearance of hyperaemia over the surface of the previously ischaemic cyanotic segment. The entire surgical process was completed under a terminal anaesthetic during which anaeasthesia was fully maintained with the top-up doses of pentobarbitone sodium (40 mg/kg). At the end of the reperfusion period, animals were sacrificed using an overdose of pentobarbitone sodium (100 mg/kg, i.v.) and the heart was removed for biochemical and immunohistochemical studies. For biochemical estimations, hearts were stored in liquid nitrogen, whereas for immunohistochemistry, hearts were fixed in 10% buffer formalin.

### Biochemical studies

The hearts were removed from liquid nitrogen, weighed and a 10% homogenate of myocardial tissue was prepared in ice-chilled phosphate buffer (50 mM, pH 7.4), and an aliquot was used to estimate thiobarbituric acid reactive substance (TBARS) according to the method described by Okhawa [16], and reduced glutathione (GSH) content was measured by the method published by Moron et al. [17]. The homogenate was centrifuged at 5000 rpm for 20 min at 4°C, and the supernatant was assayed for lactate dehydrogenase (LDH), catalase, and superoxide dismutase (SOD) activity, as well as protein content. Creatine kinase-MB (CK-MB) isoenzyme was estimated spectrophotometrically using a kit from Logotech, India whereas, TNF-α was estimated spectrophotometrically using an ELISA kit (Gen-Probe Diaclone SAS, France, UK).

### Determination of the Infarct Size

At the end of the reperfusion period, monastral blue (0.5 ml/kg) was injected into the left atrium over 30 s to determine the *in vivo* area at risk as described by Singh et al. (2006) and described in our previous study [7]. Thereafter, animals were sacrificed, and their heart was excised and left ventricle was separated and kept at −20°C for 30 min for uniform sectioning. In order to visualize the infarction, both masses (unstained and stained with blue dye) of the slices were incubated separately in 1% buffered TTC pH 8.5 for 20 min at 37°C [18].

### Histological Evaluation

As described in our previous studies [7], [19], tissues fixed in buffered formalin were embedded in paraffin, and serial sections (3 µm thick) were cut using microtome (Labindia Instruments Pvt. Ltd, Chennai, India). Each section was stained with haematoxylin and eosin (H&E). Sections were examined under the light microscope (Nikon, Tokyo, Japan), and photographs were taken. The pathologist performing microscopy was blind to the treatment status of the test subject.

### Ultrastructural Studies by Transmission Electron Microscope

As described in [7], tissue fixed with Karnovsky's solution were washed in phosphate buffer (0.1 M, pH 7.4, 6°C) and post fixed for 2 h in 1% osmium tetroxide in the same buffer at 4°C. The specimens were then washed with phosphate buffer, dehydrated with graded acetone and embedded in Araldite CY212 to make tissue blocks. Semithin (1 µm) and ultrathin sections (70–80 nm) were cut with an ultramicrotome (Ultracut E, Reichert, Austria). Sections were stained with uranyl acetate and lead acetate, and examined under a transmission electron microscope (TEM; Morgagni 268D, Fei, the Netherlands) operated at 60 kV by a morphologist blind to the status of the groups studied.

### Determination of myocardial apoptosis

#### Immunostaining for the localization of Bax and Bcl-2 proteins

Indirect immune peroxidase staining was performed as described by Mohanty et al. [20] with some modifications. Briefly, tissue sections (4-µm thickness) mounted on poly-L-lysine-coated glass slides were deparaffinised with xylene. After washing in graded concentrations of ethanol, the specimens were incubated with methanol containing 2% H_2_O_2_ to inhibit endogenous peroxidase activity. The slides were then washed with PBS for five minutes. The sections were incubated with blocking buffer (10% normal goat serum) for 10 minutes at room temperature. Next, the slides were incubated overnight at 4°C with primary mouse Bax and Bcl-2 monoclonal antibody (1∶500). For the negative control, the primary antibody was omitted. After washing with PBS, tissue sections were incubated for one hour with biotinylated goat secondary antibodies (anti-mouse IgG, 1∶100). Subsequently, sections were washed and incubated for 30 minutes with peroxidase-conjugated streptavidin-biotin complex. The target protein (Bax/Bcl-2) was visualised by incubation in peroxidase substrate (H_2_O_2_) using 3,3′-diaminobenzidine as the chromogen and the sections were counterstained with haematoxylin. Myocardial Bax and Bcl-2 proteins were quantitatively analyzed.

TUNEL assays were performed using a cell death detection kit (Roche, Indianapolis, IN, USA) as described by Mohanty et al. [20] and according to the manufacturer's instructions. The percentage of positive apoptotic cells was determined based on the number of positively stained apoptotic myocytes/total number of myocytes ×100.

### Western Blot Analysis

Western blot analysis was performed as described in our previous study (Goyal et al., 2011). Briefly, heart tissues (40 µg protein samples) were separated by SDS-PAGE, transferred to nitrocellulose membrane which was blocked for 2 h with 5% bovine serum albumin and incubated for 12 h at 4°C with a rat primary antibody (β-actin and PPAR-γ). The primary antibody was detected with horse radish peroxidase-conjugated secondary antibody. The blots so obtained were scanned and densitometry was performed to quantify the expression of β-actin (primary1: 1000 and secondary goat anti rabbit 1: 2000) and PPAR-γ (primary 1: 1000 and secondary goat anti mouse 1: 5000) using Bio-Rad Quantity One 4.4.0 software (Bio-Rad, Hercules, CA, USA).

### Statistical analysis

All numerical data in figures are expressed as the mean±SEM. Two-way analysis of variance (ANOVA) was applied for statistical analysis with *post hoc* Bonferroni's multiple comparison test and one way ANOVA for western blot analysis. Statistical value p<0.05 was considered significant.

## Results

### Mortality

An overall mortality of 15% was observed during the study period. The animals were lost because of severe diabetes, bleeding or occlusion of the coronary artery during surgery. The groups which were affected with mortality of animals were IR-SHAM (1 animal), IR-Control (3 animals), IR-GW9662 (3 animals) and IR-GW9662-Hesperidin (2 animal). There was a loss of total nine animals due to mortality and these animals were not considered for statistical analysis.

### Haemodynamic Parameters

**Figure 2:**
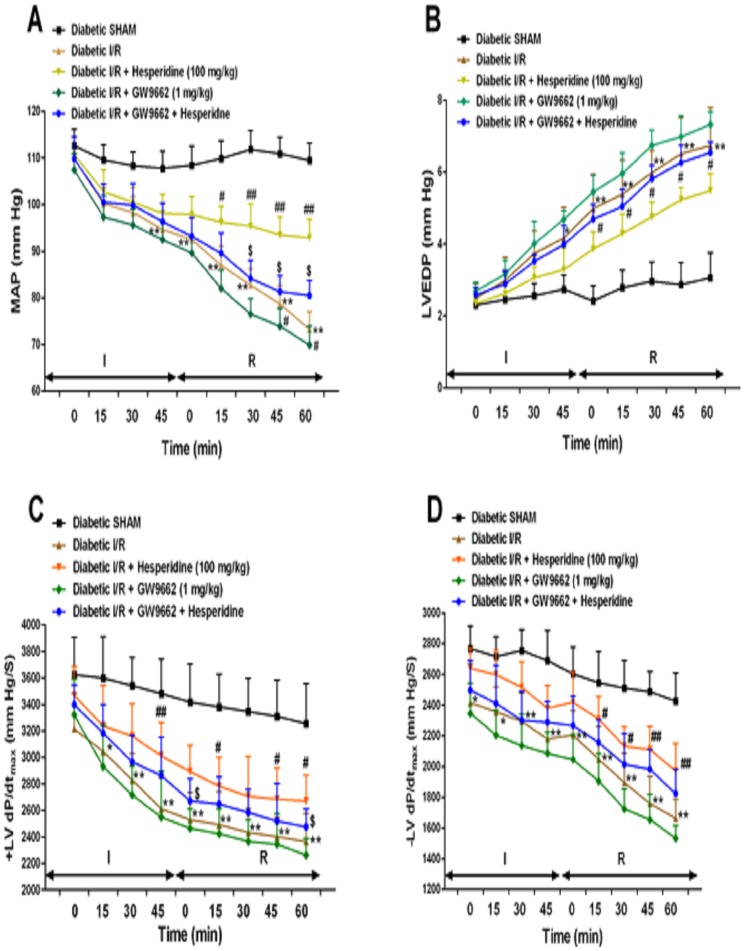
Effect of hesperidin and GW9662 on mean arterial pressure (MAP) and left ventricular (LV) function following ischaemia/reperfusion (I/R) in diabetic rats. (A) MAP, (B) left ventricular end-diastolic pressure (LVEDP), (C) maximal positive rate of left ventricular pressure (+LVdP/dtmax) and (D) maximal negative rate of −LVdP/dtmax. Data are expressed as the mean ± standard error (n = 22/group). Significance was determined by repeated measures analysis of variance followed by the Bonferroni's post hoc test: *p<0.05, **p<0.001 vs Diabetic SHAM; #p<0.05, ##p <0.001 vs Diabetic I/R; $p<0.01 vs respective Hesperidin.

The effects of hesperidin and GW9662 on haemodynamic and LV functions during I/R-induced MI in diabetic rats are depicted in Figure 2 (A–D). While significant decrease (p<0.001) was observed in the MAP and ±LVdP/dtmax, increased LVEDP was recorded in the rats subjected to cardiac I/R as compared to diabetic sham group. Fourteen days pretreatment with hesperidin significantly increased MAP, ±LVdP/dtmax and decreased LVEDP throughout I/R period at every time point, as compared to the diabetic I/R group. The dose of hesperidine (100 mg/kg/day for 14 days) was selected depending on the dose dependent study of hesperidine (data not shown). Hesperidine at 100 mg/kg produced significant results as compared to that of 50 and 200 mg/kg. Thus, 100 mg/kg dose was finalized for the entire work and administered to animals to study the effect of cardioprotection in rats. On the other hand pretreatment with GW9662 showed opposite effects as that of hesperidin and worsen the conditions as compared to that in the diabetic I/R group. Interestingly, pretreatment with GW9662 fifteen min prior to hesperidin significantly blocked the reversal effects of hesperidin in the diabetic I/R conditions for MAP, ±LVdP/dtmax and LVEDP throughout I/R period at every time point as compared to respective hesperidin treatment.

### Biochemical Parameters

As shown in Table 1, the activities of the CK-MB isoenzyme, LDH, SOD, catalase and GSH content had declined significantly (p<0.0001) in the diabetic I/R group, whereas the lipid peroxidation (p<0.0001) and TNF-*α* (p<0.0001) levels were markedly increased in the myocardium as compared to diabetic sham group. Hesperidin treatment for 14 days in diabetic I/R rats significantly (p<0.0001) reversed the activities of the CK-MB isoenzyme and LDH, and increased the content of these antioxidants in comparison to the diabetic I/R group. In addition, the hesperidin treatment significantly reduced the level of MDA (p<0.0001) and prevented the TNF-*α* increase (p<0.0001). In contrast to hesperidin, GW9662 treatment for 14 days produced the opposite effects and when administered 15 min prior to hesperidin in additional group of rats, it significantly attenuated the cardioprotective effects of hesperidin.

**Table 1:** Effect of hesperidin and GW9662 on cardiac injury marker enzymes, lipid peroxidation, TNF-α and antioxidant parameters.

Parameters	Diabetic sham	Diabetic I/R	Diabetic I/R + Hesperidin	Diabetic I/R + GW9662	Diabetic I/R + GW9662 + Hesperidin
CK-MB (IU/mg protein)	130.19±5.08	89.54±9.16*	126.55±7.89^#^	93.47±2.98	103.54±7.19^@^
LDH (IU/mg protein)	82.33±4.89	59.84±3.96*	76.59±6.15^#^	47.62±5.49	64.29±4.01^@^
MDA (nmol/g tissue)	34.86±6.21	62.31±6.19*	41.94±4.06^#^	60.84±5.97	52.37±5.91^@^
SOD (U/mg protein)	12.64±3.06	7.19±2.06*	10.86±3.19^#^	4.85±1.21	6.95±1.35^@^
CAT (U/mg protein)	48.84±6.22	31.97±5.26*	44.61±5.9^#^	24.41±2.19	39.28±4.64^@^
GSH (µg/g tissue)	4.28±0.54	1.59±0.81*	3.84±0.67^#^	1.85±0.41	2.98±0.78^@^
TNF-α (Pg/mg protein)	58.34±1.96	119.86±6.57*	78.94±6.19^#^	132.67±7.56	91.48±6.34^@^

Data are expressed as the mean ± standard error (n = 6/group). CAT, catalase; CK-MB, creatine kinase-MB isoenzyme; GSH, reduced glutathione; I/R, ischaemia/reperfusion; LDH, lactate dehydrogenase; MDA, malondialdehyde; SOD, superoxide dismutase; TNF-α, tumour necrosis factor-alpha. *p<0.001 vs Diabetic Sham; #p<0.001 vs Diabetic I/R; @p<0.01 vs Diabetic I/R + Hesperidin.

### Evaluation of the Infarct Size

A major predictor of infarct size in models of regional ischaemia is determination of percent mean area at risk and infarct area. Both the parameters were evaluated in diabetic I/R induced rats in the present study. Chronic hesperidin treatment depicted the significantly decreased infarct area (p<0.01) as compared to diabetic I/R group. However, the GW9662 treatment in the diabetic I/R group showed significantly greater infarct area (60.81±5.64, p<0.001) as compared to that in the diabetic I/R rats. Furthermore, GW9662 treatment prior to hesperidin also attenuated the cardioprotective effects of hesperidin (p<0.05) as compared to that of the hesperidin (Table 2).

**Table 2:** Mean area at risk and infarct area in rats in the different experimental groups.

Groups	Mean area at risk (%)	Infarct area (%)
Diabetic I/R	41.68±5.49	51.49±4.65
Diabetic I/R + Hesperidin	37.43±4.91	42.64±6.18*
Diabetic I/R + GW9662	44.29±5.88	60.81±5.64**
Diabetic I/R + GW9662 + Hesperidin	40.05±6.06	46.24±6.98^#^

Data are expressed as the mean ± standard error (n = 6/group). I/R, ischaemia/reperfusion. *p<0.05, **p<0.01 vs Diabetic I/R; ^#^p<0.01 vs Diabetic I/R + Hesperidin.

### Histopathological evaluation

**Figure 3:**
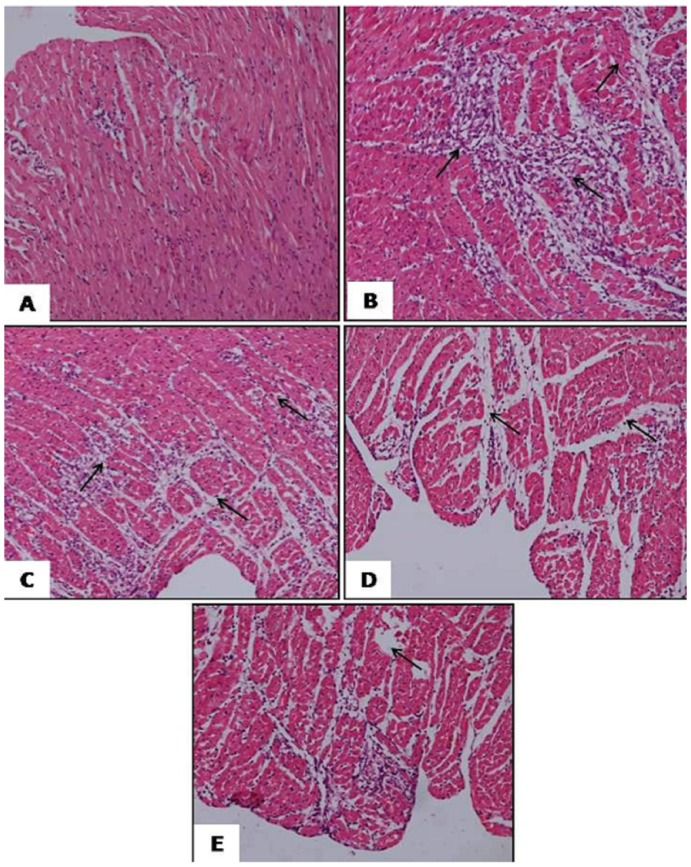
Photomicrograph showing the histopathological changes in the rat myocardium. (A) represents haematoxylin and eosin staining [(H&E) ×200] in diabetic sham group, B diabetic ischaemia/reperfusion (I/R) group (H&E ×200), (C) diabetic I/R + hesperidin treatment (100 mg/kg) (H&E ×200), (D) diabetic I/R + GW9662 (1 mg/kg) (H&E ×200) and (E) diabetic I/R rats + GW9662 + hesperidin (H&E ×200).

Table 3 and Figure 3 summarizes the histopathological changes following the saline, hesperidin or GW9662 treatments either alone in combination in the diabetic I/R induced rats. While diabetic sham group did not show any inflammation, oedema and necrosis (Figure 3A), myocardial membrane damage with extensive myonecrosis, fibroblastic proliferation, inflammatory cell infiltration and marked oedema were significantly observed in the diabetic I/R induced rats (Figure 3B). On contrary, hesperidin pre treatment in the diabetic I/R rats showed the occasional areas of myofibre loss with necrosis but no oedema and inflammatory cell infiltration (Figure 3C). However, the degree of myocardial damage in the GW9662 pretreated rats was severe as compared to diabetic I/R rats (Figure 3D). Interestingly, the rats pretreated with GW9662 previous to hesperidin showed attenuation in the effects of hesperidin (Figure 3E).

**Table 3:** Histopathological changes in all experimental groups.

	Myonecrosis	Edema	Inflammation
Diabetic Sham	−	−	−
Diabetic I/R	+++	+++	+++
Diabetic I/R + Hesperidin	+	−	−
Diabetic I/R + GW9662	++++	++++	++++
Diabetic I/R + GW9662 + Hesperidin	++	+	+

I/R, ischaemia/reperfusion. Score (−), absence of any myonecrosis, oedema and inflammation; score (+), focal areas of myonecrosis, oedema and inflammation; score (++), patchy areas of myonecrosis, oedema and inflammation; score (+++), confluent areas of myonecrosis, oedema and inflammation; score (++++), massive areas of myonecrosis, oedema and inflammation.

### Ultrastructural Results

**Figure 4:**
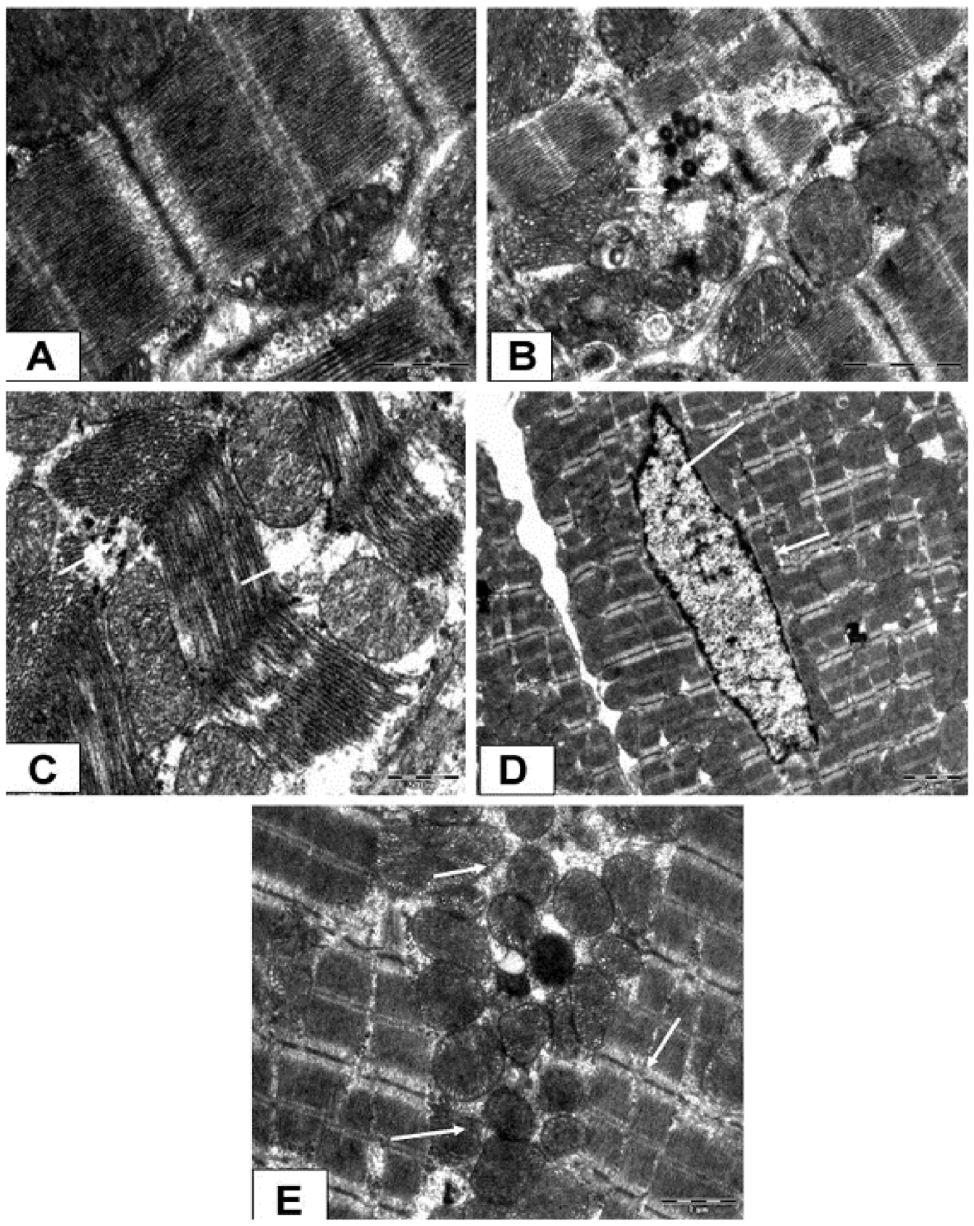
Photomicrograph showing the ultrastructural changes in the rat myocardium. (A) represents transmission electron microscope [(TEM) ×4800] in diabetic sham group, B diabetic ischaemia/reperfusion (I/R) group (TEM ×3500), (C) diabetic I/R + hesperidin treatment (100 mg/kg) (TEM ×3500), (D) diabetic I/R + GW9662 (1 mg/kg) (TEM ×3500) and (E) diabetic I/R rats + GW9662 + hesperidin (TEM ×3500).

As shown in Figure 4A, diabetic sham rats showed well preserved mitochondrial structure and glycogen granules throughout the myocardium. Significant disruption of the myofibrils, myonecrosis, depletion of the glycogen reserves, swollen and irregular mitochondria with a loss of cristae and chromatin condensation was observed in the diabetic I/R rats (Figure 4B). On the other hand, hesperidin treatment showed only a mild separation of the mitochondrial cristae without swelling and vacuolation (Figure 4C). More severe ultrastructural complications in conjunction with severe peripheral nuclear condensation were observed in the GW9662-treated group as compared to diabetes I/R rats (Figure 4D). In addition, GW9662 treatment prior to hesperidin blocked the recovery process of hesperidin *per se* (Figure 4E).

### Effect of Hesperidin or GW9662 alone or in combination on expression of Bcl-2 and Bax proteins in experimentally induced MI

**Figure 5:**
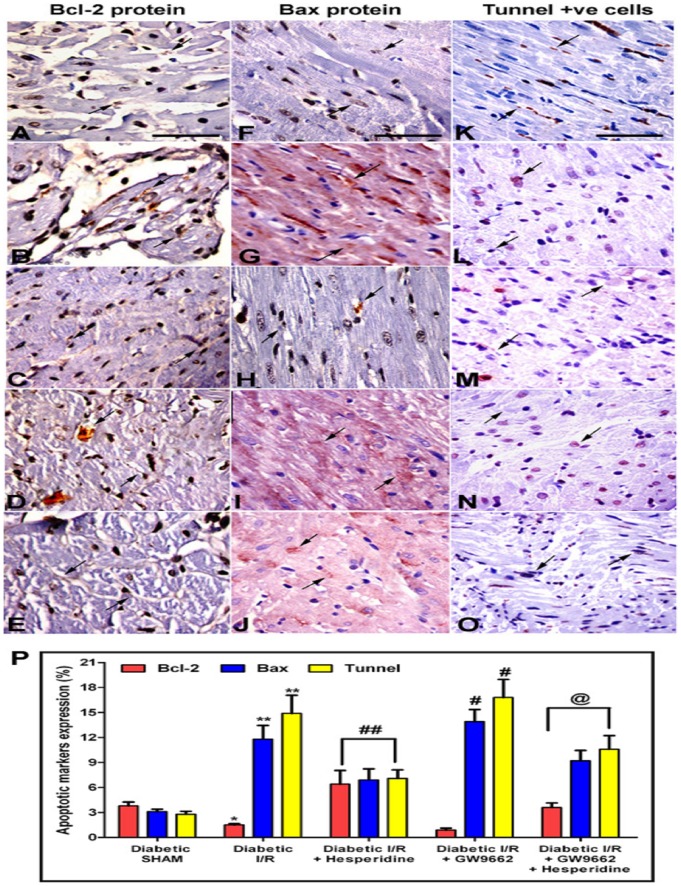
Photomicrograph of myocardial tissue sections showing Bcl-2 (A–E), Bax proteins expression (F–J) and TUNEL-positive cells (K–O). Apoptotic nuclei are indicated by arrows in panels (×400). (A, F and K) represents Diabetic sham rats treated with vehicles, (B, G and L) diabetic ischaemia/reperfusion (I/R) rats treated with vehicles, (C, H and M) diabetic I/R rats treated with hesperidin, (D, I and N) diabetic I/R rats treated with GW9662 and (E, J and O) diabetic I/R rats treated with GW9662 and hesperidin. (P) represents the quantitative analysis of the Bcl-2, Bax proteins and TUNEL-positive cells in the different groups. Data are expressed as the mean ± standard error (n = 6). *p<0.05, *p<0.001 vs diabetic SHAM; #p<0.05, ##p<0.001 vs Diabetic I/R group; @p<0.01 vs Diabetic I/R + hesperidin.

To confirm the involvement of PPAR-γ receptors in the cardioprotection by Hesperidin, the estimation of apoptosis regulatory proteins (Bax and Bcl-2) using immunohistopathological studies were conducted in the diabetic sham and diabetic I/R rats treated with vehicle, hesperidin and GW9662 either alone or in combination. Photomicrographs in the Figure 5 (A-E), (F-J) and (K-O) shows the effect of hesperidin and GW9662 either alone or in combination on Bcl-2, Bax proteins and TUNEL positive cells, respectively, in the diabetic I/R rats and Figure 5P represents the quantitative analysis of the same. While significant decrease in Bcl-2 was observed (B), Bax proteins and TUNEL positive cells were significantly increased in the diabetic I/R rats (G and L) as compared to diabetic sham control (A, F and K). On the other hand, hesperidin treatment significantly prevented the changes in Bcl-2 and Bax proteins and TUNEL positive cells as observed in the Diabetic I/R group (C, H and M). In contrast to this, GW9662 treatment exaggerated the diabetic I/R induced conditions (D, I and N), and also attenuated the hesperidin induced effects with respect to the Bcl-2 and Bax proteins, and TUNEL positive cells (E, J and O).

### Western Blot Analysis

**Figure 6:**
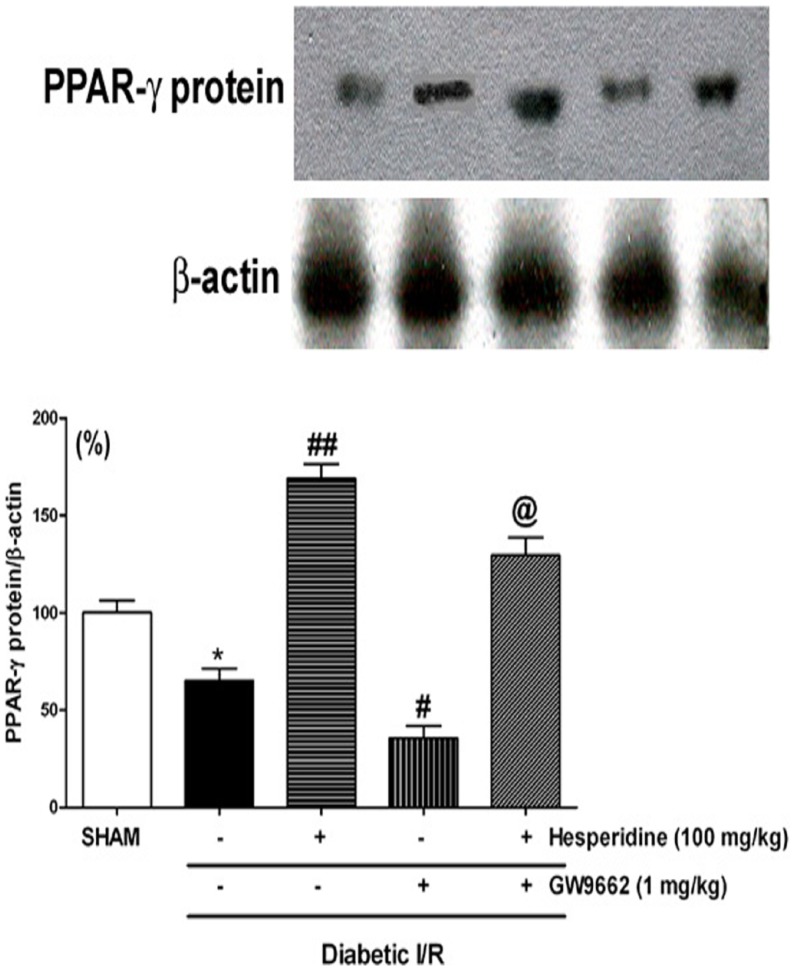
Effect of hesperidin and GW9662 (PPAR-γ antagonist) on PPAR-γ expression (top) in ischaemia/reperfusion (I/R)-induced myocardial infarction in diabetic rats. The bar graphs show PPAR-γ protein expression that has been normalized to β-actin (bottom). Data are expressed as a ration of the value determined for the vehicle (diabetic sham) (set as 100%). Data are expressed as the mean ± standard error. *p<0.05 vs Diabetic SHAM; #p<0.01, #p<0.001 vs Diabetic I/R; @p<0.05 vs Hesperidin.

The expression of PPAR-γ was carried out in diabetic sham and I/R rats and those induced diabetic I/R and treated with hesperidin and GW9662 alone or in combination (Figure 6). The expression of PPAR-γ was significantly lower (p<0.05) in the diabetic I/R rats as compared to that of the sham rats. Moreover, the hesperidin treated rats showed increased expression of PPAR-γ protein (p<0.001) as compared to that of the diabetic I/R group. On the other hand, GW9662 treatment not only significantly decreased (p<0.05) the protein expression but also significantly attenuated (p<0.05) hesperidin induced increase in the PPAR-γ expression.

## Discussion

The present study for the first time evidenced the involvement of PPAR-γ pathway in the cardioprotective activity of hesperidin in the cardiac I/R model in diabetic rats. Herein, streptozotocine was used for the induction of diabetes in rats, since it enters the β-cells in pancreas and alkylates DNA. This depletes NAD+ and ATP and aggravates the diabetogenic effect by activating poly ADP-ribosylation pathway. In addition to this the activation of reactive oxygen species (ROS), superoxide and hydroxy radicals augments cytotoxic action. These results into destruction of β-cells and also cause injury to myocardial tissues leading to ischemia like conditions. Myocardial injury can be experimentally induced in rats using I/R model which mimics the human pathophysiological condition and reduces arterial pressure along with ventricular dysfunction [19], [21]. Blockade or occlusion of LAD results in myocardial ischemia which includes complex series of cellular events and lead to myocardial cell death, furthermore, reperfusion (restoration of blood flow) enhances myocardial injury and diminishes cardiac contractile functions and metabolic derangements. Cardiac I/R induced group of rats depicted significantly impaired systolic and diastolic functions (decrease in MAP) and inotropic and lusitropic state (±LVdP/dtmax), and increased ventricular remodeling (preload LVEDP). Further, this impairment was confirmed by pathological assessment as characterized by inflammatory cells, necrosis, edema, and replacement of myofibrils by interfibrillar spaces. While hesperidin treatment improved LVEDP by increasing inotropic (+LVdP/dt_max_, marker of myocardial contraction) and lusitropic (-LVdP/dt_max_, marker of myocardial relaxation) states of the heart and decreased systolic, diastolic, and mean arterial pressures, as compared to the diabetic control group, PPAR-γ antagonist GW9662 worsen the conditions. Moreover, prior treatment of GW9662 also prevented the hesperidin induced recovery in rats. PPAR-γ activation is directly correlated to the beneficial effects on all the haemodynamic parameters in spontaneously hypertensive rats [22], salt-sensitive hypertensive rats [23] and in renovascular animal model of hypertension [24]. Geng et al. [25] also found that rosiglitazone (a thiazolidinedione analogue) improves the left ventricular hemodynamic function in rats with MI due to their PPAR-γ agonist activity. Thus, these observations suggest the cardioprotective role of hesperidin in diabetic I/R model of cardiac ischemia via PPAR-γ pathway and that its activation may enhance these effects.

Further to confirm these observations we also studied the biochemical estimations in all the five groups namely sham control, diabetic I/R induced rats treated with saline, hesperidin, GW9662 either alone or in combination. We studied lipid peroxide metabolism and cardiac injury marker enzymes (CK-MB isoenzyme and LDH) and MDA, antioxidant enzymes (SOD, catalase and GSH) and TNF-α. CK-MB isoenzyme and LDH are important hallmarks of I/R injury and plays pivotal role in the pathogenesis of MI. In the present study, while decreased levels of CK-MB and LDH, increased levels of MDA were encountered in the heart of diabetic rats subjected to cardiac I/R injury which denotes oxidative stress. These observations are in accordance with the previous literature where increased lipid peroxidation products and MDA and decreased CK-MB isoenzymes and LDH was observed in hearts exposed to oxidative stress [26]. Myocardial CK-MB isoenzyme and LDH activities were decreased in the diabetic rats [27].Serum LDH activity was found to elevate in the patients with type II diabetes [28]. While, hesperidin treatment increased the levels of CK-MB, LDH and MDA levels in such cardiac I/R injury model, GW9662 treatment worsen the conditions. These observations are in accordance with the previous literature which also put forward the protective role of hesperidine in similar models [29], [30]. Moreover, prior treatment of GW9662 also prevented the hesperidin induced restoration in I/R injury. These results for the first time put forward the involvement of PPAR-γ mechanism in the activity of hesperidin in diabetic I/R injured rats. Further, the antioxidant enzymes like SOD, catalase and GSH were also studied and in addition TNF-α levels were also observed. Dhalla et al. [31] have suggested the importance of these antioxidant enzymes in the protection of myocardial cells against I/R like injury. Significant decrease in the antioxidant enzymes (SOD, catalase and GSH) was observed in the diabetic rats subjected to cardiac I/R injury. The levels were increased significantly in the hesperidin treated group thus categorizing hesperidin as a major antioxidant. In contrast, the GW9662 produced the opposite effects per se and blocked the antioxidant effects of hesperidin which suggests the involvement of PPAR-γ pathway in the hesperidin induced antioxidant effects in the cardiac I/R injury model.

The ability of hesperidin to limit the infarct size in cardiac I/R injured diabetic rats was observed. The percent infarction was significantly decreased in the rats treated with hesperidin, while GW9662 treatment *per se* increased the percent infarct area it also limited the hesperidin induced recovery when given prior to hesperidin. To support this observation TNF-α levels were studied which was found increased in the cardiac I/R injured diabetic rats. The TNF-α levels were decreased in the hesperidin treated rats and the prior treatment of GW9662 prevented this decrease. It seems that, hesperidine treatment may inhibit the TNF-α production, which is known to be one of the major factor in inflammation and infarction [7]. Since, in previous studies PPAR-γ agonists such as rosiglitazone and pioglitazone have been shown to reduce the infarct size following I/R injury [32], and in present study prior treatment of PPAR-γ antagonist attenuated the hesperidin induced recovery, it proves the role of PPAR-γ mechanism in hesperidine induced responses.

In addition to above parameters cardiac apoptosis was also studied, which is suggested to be one of the major pathogenic causes that underlie myocardial I/R injury. Crow et al. [33] suggested that blocking the apoptosis process might help in the prevention of loss of contractile cells, decrease the I/R induced cardiac injury and thus prevent the occurrence of MI. With this information in mind and with a view to explore the underlying mechanisms responsible for the improvement of cardiac functions following hesperidin treatment, the levels of antiapoptotic protein Bcl-2 and the proapoptotic protein Bax were measured and TUNEL staining was performed which is a known marker of DNA fragmentation. Cardiac I/R injury in diabetic rats significantly decreased the Bcl-2 and increased BAX proteins and TUNEL-positive cells. Moreover, hesperidin treatment in these diabetic cardiac I/R injured rats showed significant antiapoptotic potential as depicted by augmented Bcl-2 protein expression and decreased Bax protein expression and TUNEL-positive cells. However, GW9662 treatment worsen the conditions in the diabetic I/R injured rats and its prior treatment attenuated the hesperidin induced improvement. The involvement of PPAR-γ mechanism in the antiapoptotic activity is much well known. PPAR-γ agonists, rosiglitazone and pioglitazone, markedly decreased apoptosis in the hearts of rabbits with hypercholesterolaemia and rats subjected to myocardial I/R injury [34], [35]. Thus, the attenuation of the hesperidin induced improvement of cardiac functions in the presence of GW9662 (PPAR-γ antagonist) confirms the involvement of PPAR-γ pathway in the hesperidin mediated actions in diabetic cardiac I/R injury.

With a view to confirm the rescuing effects of hesperidin in the diabetic I/R injury model light microscopy and ultrastructural studies were carried out in sham rats, rats subjected to diabetic I/R injury and treated with saline, hesperidin or GW9662 either alone or in combination. Hesperidin treatment in I/R injured diabetic rats showed the protection of normal morphology of myocardium with no infarction, which was impaired in I/R injury control. Similarly, the ultrastructural studies also depicted the cardioprotective effects of hesperidin. On the other hand, GW9662, PPAR-γ antagonist, treatment showed severe infarction of the myocardial fibres, oedema and increased inflammatory cells. Moreover, the prior treatment of GW9662 with hesperidin also attenuated the hesperidin induced improvement. In addition, the western blot analysis depicted the increased expression of PPAR-γ protein, whereas GW9662 significantly decreased the concentration of PPAR-γ expressed *per se* and attenuated hesperidin induced expression. Previous studies also underscore the role PPAR-γ pathway in the different animal models [36], [37]. Thus, the observations confirms the involvement of PPAR-γ mediated mechanisms in the I/R injury model in diabetic rats. Several additional confirmative studies may be required for further strengthening of this hypothesis. Limited bioavailability of hesperidin is reported in human volunteers, since low plasma concentration of hesperetin aglycone (<2 µmol/L) was observed after ingesting 0.5–1 L of orange juice [38], [39]. Hesperidin is hydrolyzed by β-glucosidase derived from gut microflora into hesperetin when ingested orally, and its conjugated metabolites such as hesperetin glucuronides and sulfoglucuronides are absorbed into the blood in humans and rats [39]–[41]. However, no free aglycone is detected in the circulating blood. In human plasma the maximum concentrations of hesperetin metabolites following 5–7 h of orange juice ingestion are 1.3–2.2 mM for intake of 130–220 mg [42]. Amongst hesperetin metabolites, hesperetin-7-O-glucuronide and hesperetin-30-Oglucuronide are detected as the predominant metabolites in rat plasma following oral administration of hesperidin (50 mg/kg) [41]. Hesperetin-7-O-glucuronide also exert hypotensive, vasodilatory and anti-inflammatory activities [43]. Thus, the administration of PPAR-γ antagonist may prolong the activity of hesperidin metabolites and produce cardioprotective activity. Taken together, hesperidin treatment reduces oxidative stress and increases blood flow in ischaemic regions of the heart by correcting the I/R induced cardiac dysfunction in diabetic rats. Prior treatment with PPAR-γ antagonist attenuated the effects of hesperidine. Thus, the study for the first time proves the pivotal role of PPAR-γ mechanisms in the hesperidin induced cardioprotective effects in the I/R injury model in diabetic rats. The activation of PPAR-γ pathway along with the hesperidin like agents in the I/R injury like conditions may serve therapeutic importance in diabetic patients.

## References

[1] Lopez AD, Murrau CC (1998) The global burden disease, 1990–2020. Nat Med 4: 1241–1243. 9809543 10.1038/3218

[2] Bibbins-Domingo K, Coxson P, Pletcher MJ, Lightwood J, Goldman L (2007) Adolescent overweight and future adult coronary heart disease. N Engl J Med 6: 2371–2379. 10.1056/NEJMsa073166 18057339

[3] De Bono DP, Boon NA (1992) Diseases of the cardiovascular system. In: Edwards CRW, Boucheir IAS, editors. Davidson's Principles and Practice and Medicine. Churchill Livingstone, Hong Kong, pp. 249–340.

[4] DeBiase L, Pignatelli P, Lenti L, TocciG, Piccioni F, et al (2003) Enhanced TNF alpha and oxidative stress in patients with heart failure: effect of TNF alpha on platelet O2- production. Thromb Haemost 90: 317–325. 12888880 10.1160/TH03-02-0105

[5] Rajadurai M, Prince PSM (2006) Preventive effect of naringin on lipid peroxides and antioxidants in isoproterenol induced cardiotoxicity in Wistar rats: Biochemical and histopathological evidences. Toxicology 228: 259–268. 17084010 10.1016/j.tox.2006.09.005

[6] Nakamura T, Nishi H, Kokusenya Y, Hirota K, Miura Y (2000) Mechanism of antioxidative activity of fluvastatin-determination of the active position. Chem Pharm Bull 48: 235–237. 10705511 10.1248/cpb.48.235

[7] Goyal SN, Bharti S, Bhatia J, Nag TC, Ray R, et al (2011) Telmisartan, a dual ARB/partial PPAR-γ agonist, protects myocardium from ischaemic reperfusion injury in experimental diabetes. Diabetes Obes Metab 13: 533–541. 21320264 10.1111/j.1463-1326.2011.01377.x

[8] Park HJ, Kim MJ, Ha E, Chung JH (2008) Apoptotic effect of hesperidin through caspase3 activation in human colon cancer cells, SNU-C4. Phytomedicine 15: 147–151. 17897817 10.1016/j.phymed.2007.07.061

[9] Wilmsen PK, Spada DS, Salvador M (2005) Antioxidant activity of the flavonoid hesperidin in chemical and biological systems. J Agric Food Chem 53: 4757–4761. 15941311 10.1021/jf0502000

[10] Gumieniczek A (2003) Effect of the new thiazolidinedione-pioglitazone on the development of oxidative stress in liver and kidney of diabetic rabbits. Life Sci 74: 553–562. 14623026 10.1016/j.lfs.2003.03.004

[11] Salam NK, Huang TH, Kota BP, Kim MS, Li Y, et al (2008) Novel PPAR-gamma agonists identified from a natural product library: a virtual screening, induced-fit docking and biological assay study. ChemBiol Drug Des 71: 57–70. 10.1111/j.1747-0285.2007.00606.x 18086153

[12] VandenHeuvel JP (1999) Peroxisome proliferator-activated receptors (PPARS) and carcinogenesis. Toxicol Sci 47: 1–8. 10048147 10.1093/toxsci/47.1.1

[13] Hihi AK, Michalik L, Wahli W (2002) PPARs: transcriptional effectors of fatty acids and their derivatives. Cell Mol Life Sci59: 790–798. 10.1007/s00018-002-8467-x PMC11146119 12088279

[14] Ondrey F (2009) Peroxisome proliferator-activated receptor gamma pathway targeting in carcinogenesis: implications for chemoprevention. Clin Cancer Res 15: 2–8. 19118026 10.1158/1078-0432.CCR-08-0326

[15] Ito H, Nakano A, Kinoshita M, Matsumori A (2003) Pioglitazone, a peroxisome proliferator-activated receptor-gamma agonist, attenuates myocardial ischemia/reperfusion injury in a rat model. Lab Invest 83: 1715–1721. 14691289 10.1097/01.lab.0000106724.29121.da

[16] Okhawa H, Oohishi N, Yagi N (1979) Assay for lipid peroxides in animal tissues by thiobarbituric acid reaction. Anal Biochem 95: 351–358. 36810 10.1016/0003-2697(79)90738-3

[17] Moron MS, Depierre JW, Mannervik B (1979) Levels of glutathione, glutathione reductase and glutathione S-transferase activities in rat lung and liver. Biochem Biophys Acta 582: 67–78. 760819 10.1016/0304-4165(79)90289-7

[18] Singh AD, Amit S, Kumar OS, Rajan M, Mukesh N, et al (2006) Cardioprotective effects of bosentan, a mixed endothelin type A and B receptor antagonist, during myocardial ischaemia and reperfusion in rats. Basic Clin Pharmacol Toxicol 98: 604–610. 16700825 10.1111/j.1742-7843.2006.pto_405.x

[19] Loh HK, Sahoo KC, Kishore K, Ray R, Nag TC, et al (2007) Effects of thalidomide on isoprenaline- induced acute myocardial injury: a haemodynamic, histopathological and ultrastructural study. Basic Clin Pharmacol Toxicol 100: 233–239. 17371527 10.1111/j.1742-7843.2007.00022.x

[20] Mohanty IR, Arya DS, Gupta SK (2008) Withania somnifera provides cardioprotection and attenuates ischemia-reperfusion induced apoptosis. Clin Nutr 27: 635–642. 18620784 10.1016/j.clnu.2008.05.006

[21] Mohanty I, Gupta SK, Arya DS (2007) Anti-apoptotic and cardioprotective effects of an herbal combination in rats with experimental myocardial infarction. Int J Integr Biol 1: 178–188.

[22] Wu L, Wang R, De Champlain J, Wilson TW (2004) Beneficial and deleterious effects of rosiglitazone on hypertension development in spontaneously hypertensive rats. Am J Hypertens 17: 749–756. 15363815 10.1016/j.amjhyper.2004.04.010

[23] Bolten CW, Payne MA, McDonald WG, Blanner PM, Chott RC, et al (2007) Thiazolidinediones inhibit the progression of established hypertension in the Dahl salt-sensitive rat. DiabVasc Dis Res 4: 117–123. 10.3132/dvdr.2007.029 17654445

[24] de Oliveira Silva-Junior G, da Silva Torres T, de Souza Mendonca L, Alberto Mandarim-de-Lacerda C (2011) Rosiglitazone (peroxisome proliferator-activated receptor-gamma) counters hypertension and adverse cardiac and vascular remodeling in 2K1C hypertensive rats. Exp Toxicol Pathol 63: 1–7. 19775877 10.1016/j.etp.2009.09.001

[25] Geng DF, Wu W, Jin DM, Wang JF, Wu YM (2006) Effect of peroxisome proliferator-activated receptor gamma ligand. Rosiglitazone on left ventricular remodeling in rats with myocardial infarction. Int J Cardiol 113: 86–91. 16891009 10.1016/j.ijcard.2006.03.060

[26] CharanSahoo K, Arora S, Goyal S, Kishore K, Ray R, et al (2009) Cardioprotective effects of benazepril, an angiotensin-converting enzyme inhibitor, in an ischaemia-reperfusion model of myocardial infarction in rats. J Renin Angiotensin Aldosterone Syst 10: 201–209. 20026869 10.1177/1470320308353059

[27] Hagar HH (2002) Folic acid and vitamin B12 supplementation attenuates isoprenaline-induced myocardial infarction in experimental hyperhomocysteinemic rats. Pharmacol Res 46: 213–219. 12220963 10.1016/s1043-6618(02)00095-6

[28] Huang EJ, Kuo WW, Chen YJ, Chen TH, Chang MH, et al (2006) Homocysteine and other biochemical parameters in type 2 diabetes mellitus with different diabetic duration or diabetic retinopathy. Clin Chim Acta 366: 293–298. 16343469 10.1016/j.cca.2005.10.025

[29] Kakadiya J, Shah M, Shah N (2010) Effect of hesperidin on serum heart marker, myocardial tissues parameter and histopathological of heart in isoproterenol induced myocardial infarction in diabetic rats. Int J Biol Tech 1: 57–62.

[30] Kakadiya J, Mulani H, Shah N (2010) Protective effect of hesperidin on cardiovascular complication in experimentally induced myocardial infarction in diabetes in rats. J Basic Clin Pharm 1: 85–91. 24825971 PMC3979178

[31] Dhalla NS, Elmoselhi AB, Hata T, Makino N (2000) Status of myocardial antioxidants in ischemia-reperfusion injury. Cardiovasc Res 47: 446–456. 10963718 10.1016/s0008-6363(00)00078-x

[32] Wayman NS, Hattori Y, McDonald MC, Mota-Filipe H, Cuzzocrea S, et al (2002) Ligands of the peroxisome proliferator-activated receptors (PPAR-gamma and PPAR-alpha) reduce myocardial infarct size. FASEB J 16: 1027–1040. 12087064 10.1096/fj.01-0793com

[33] Crow MT, Mani K, Nam YJ, Kitsis RN (2004) The mitochondrial death pathway and cardiac myocyte apoptosis. Circ Res 95: 957–970. 15539639 10.1161/01.RES.0000148632.35500.d9

[34] Liu HR, Tao L, Gao E, Lopez BL, Christopher TA, et al (2004) Anti-apoptotic effects of rosiglitazone in hypercholesterolemic rabbits subjected to myocardial ischemia and reperfusion. Cardiovasc Res 62: 135–144. 15023560 10.1016/j.cardiores.2003.12.027

[35] Cao Z, Ye P, Long C, Chen K, Li X, et al (2007) Effect of pioglitazone, a peroxisome proliferator-activated receptor gamma agonist, on ischemia-reperfusion injury in rats. Pharmacology 79: 184–192. 17356310 10.1159/000100870

[36] Ikejima H, Imanishi T, Tsujioka H, Kuroi A, Kobayashi K, et al (2008) Effects of telmisartan, a unique angiotensin receptor blocker with selective peroxisome proliferator-activated receptor-gamma-modulating activity, on nitric oxide bioavailability and atherosclerotic change. J Hypertens 26: 964–972. 18398339 10.1097/HJH.0b013e3282f52c36

[37] Kobayashi N1, Ohno T, Yoshida K, Fukushima H, Mamada Y, et al (2008) Cardioprotective mechanism of telmisartan via PPAR-gamma-eNOS pathway in dahl salt-sensitive hypertensive rats. Am J Hypertens 21: 576–581. 18437150 10.1038/ajh.2008.27

[38] Erlund I, Meririnne E, Alfthan G, Aro A (2001) Plasma kinetics and urinary excretion of the flA (2001) naringenin and hesperetin in humans after ingestion of orange juice and grapefruit juice. J Nutr 131: 235–241. 11160539 10.1093/jn/131.2.235

[39] Manach C, Morand C, Gil-Izquierdo A, Bouteloup-Demange C, Remesy C (2003) Bioavailability in humans of the flavanones hesperidin and narirutin after the ingestion of two doses of orange juice. Eur J Clin Nutr 57: 235–242. 12571654 10.1038/sj.ejcn.1601547

[40] Ameer B, Weintraub RA, Johnson JV, Yost RA, Rouseff RL (1996) Flavanone absorption after naringin, hesperidin, and citrus administration. Clin Pharmacol Ther 60: 34–40. 8689809 10.1016/S0009-9236(96)90164-2

[41] Matsumoto H, Ikoma Y, Sugiura M, Yano M, Hasegawa Y (2004) Identification and quantification of the conjugated metabolites derived from orally administered hesperidin in rat plasma. Food Chem 52: 6653–6659. 10.1021/jf0491411 15479036

[42] Manach C, Scalbert A, Morand C, Rémésy C, Jiménez L (2004) Polyphenols: food sources and bioavailability. Am J Clin Nutr 79: 727–747. 15113710 10.1093/ajcn/79.5.727

[43] Yamamoto M, Jokura H, Hashizume K, Ominami H, Shibuya Y, et al (2013) Hesperidin metabolite hesperetin-7-O-glucuronide, but not hesperetin-30-O-glucuronide, exerts hypotensive, vasodilatory, and anti-inflammatory activities. Food Funct 4: 1346–1351. 23831969 10.1039/c3fo60030k

